# Inverse Photoemission Spectroscopy of Coinage Metal Corroles: Comparison with Solution-Phase Electrochemistry

**DOI:** 10.1021/acsorginorgau.4c00027

**Published:** 2024-06-19

**Authors:** Luca Giovanelli, Younal Ksari, Hela Mrezguia, Eric Salomon, Marco Minissale, Abraham B. Alemayehu, Abhik Ghosh

**Affiliations:** † Aix-Marseille Université, CNRS, IM2NP, Marseille 13397, France; ‡ Aix-Marseille Université, CNRS, PIIM, Marseille 13397, France; § Department of Chemistry, UiT − The Arctic University of Norway, N-9037 Tromsø, Norway

**Keywords:** photoemission, photoelectron, inverse photoemission, corrole, copper, silver, gold

## Abstract

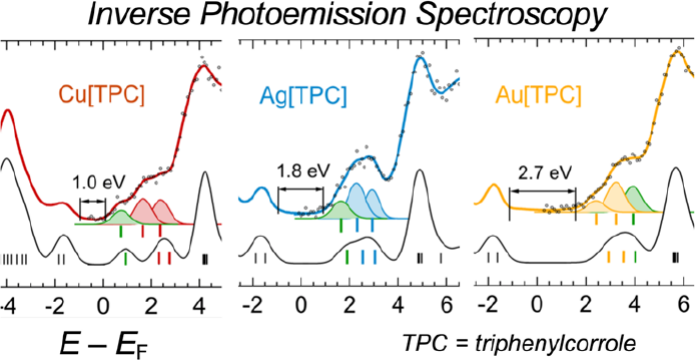

A combined direct and inverse photoemission study of coinage metal corroles suggests that the latter technique, in favorable cases, can provide some additional information relative to electrochemical measurements. Thus, whereas inverse photoemission spectroscopy (IPES) provides relative electron affinities for electron addition to different unoccupied orbitals, electrochemical reduction potentials shed light on the energetics of *successive* electron additions. While all three coinage metal triphenylcorrole (TPC) complexes exhibit similar ionization potentials, they exhibit dramatically different inverse photoemission spectra. For Cu[TPC], the lowest-energy IPES feature (0.74 eV) is found to be exceedingly close to the Fermi level; it is significantly higher for Ag[TPC] (1.65 eV) and much higher for Au[TPC] (2.40 eV). These differences qualitatively mirror those observed for electrochemical reduction potentials and are related to a partially metal-centered LUMO in the case of Cu- and Ag[TPC] and a fully corrole-based LUMO in the case of Au[TPC]; the latter orbital corresponds to the LUMO+1 in the case of Ag[TPC].

The last quarter-century has seen corroles catapulted from relative obscurity to the forefront of chemical and materials sciences and wide-ranging medical and technological applications.^1,2^ Redox-active metallocorroles, thus, are widely used as catalysts, especially as electro- and photo catalysts.^3−6^ Redox-innocent metallocorroles, especially those involving 5d transition elements, hold particular promise for medicine, perhaps most notably as triplet photosensitizers for oxygen sensing and photodynamic and related therapies.^7−11^ The new applications have built directly on an ever-deepening understanding of metallocorroles’ electronic structure, at the center of which, arguably, lies the phenomenon of ligand noninnocence.^12−14^ Noninnocent ligands, it may be recalled, do not allow a straightforward determination of the oxidation state of a coordinated metal.^15,16^ A tricky concept, the phenomenon can nonetheless be probed by a battery of physical and quantum chemical methods; very recently, the phenomenon has even been quantified.^17,18^ Presented herein is a first exploration of the potential application of inverse photoemission spectroscopy (IPES) to corrole derivatives.

Inverse photoemission spectroscopy,^19−22^ the time-reversed counterpart of direct photoemission spectroscopy^23−26^ (also called photoelectron spectroscopy, PES), plays an important role in study of the unoccupied states of materials. In a typical experiment, a monochromatic electron beam impinges on a surface, resulting in the emission of photons whose energies are analyzed. The incident electrons couple to unoccupied states of the material and decay to lower states via both radiative and nonradiative pathways and the energies of the radiated photons provides a map of the unoccupied state architecture. In a simpler implementation of the experiment, the so-called isochromat mode, the energy of the incident electrons (*E*_i_) is varied, while photons are detected at a fixed energy (*h*ν), with a narrow bandpass on the order of a 100 meV. Regardless of the implementation, the energy of the final state (*E*_f_) is given by *E*_f_ = *E*_i_ - *h*ν. Together, direct (UPS) and inverse photoemission spectroscopy (IPES) provide a picture of the band structure of a material.^27^

Unlike PES, IPES has enjoyed relatively few applications in molecular chemistry.^28−34^ Simpler tools such as electrochemistry and optical spectroscopy have typically afforded the necessary insight into molecular excited states. That said, IPES does provide unique insight. While electrochemical reduction potentials afford information on the energetics of *successive* reductions (i.e., electron additions), IPES probes the energetics of individual unoccupied molecular orbitals (MOs). To determine the potential usefulness of IPES in a coordination chemistry context, we carried out a direct and inverse photoemission study of coinage metal *meso*-triphenylcorrole complexes, M[TPC] (M = Cu, Ag, Au; Scheme 1). The solution-phase reduction potentials of the three complexes vary from −0.20 V for Cu[TPC] through −0.86 V for Ag[TPC] to −1.38 V for Au[TPC] (all vs the saturated calomel electrode), indicating dramatically rising energies of the lowest unoccupied MO (LUMO) from Cu through Ag to Au.^35−37^ The electronic structures of the complexes also vary from a noninnocent Cu^II^–Cor^•2-^ description for Cu[TPC]^38−44^ to an essentially innocent M^III^-Cor^3–^ description for Ag[TPC]^37^ and Au[TPC];^45−53^ these differences are schematically summarized in Figure 1. We shall see that the IPES-derived picture of unoccupied states is eminently consistent with that derived from electrochemistry^35−37,54^ and other spectroscopic methods (such as X-ray absorption spectroscopy^55,56^). Moreover, combining the UPS and IPES data gives access to the solid-state band gap (*E*_g_),^57^ a value that can be compared to the electrochemical HOMO–LUMO gap (*E*_ox-red_), which is the difference between solution-phase oxidation and reduction potentials obtained from electrochemical measurements.^58^

**Scheme 1:**
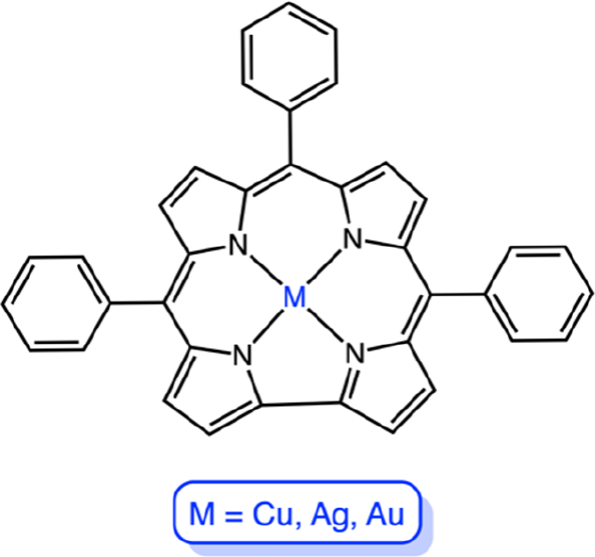
Complexes Studied in This Work: M[TPC] (M = Cu, Ag, Au)

**Figure 1:**
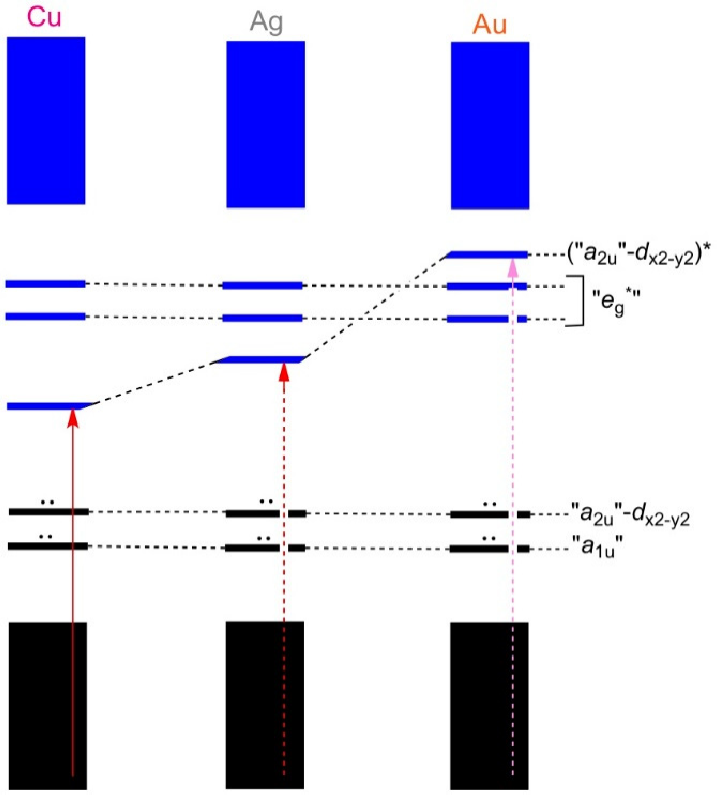
Schematic energy level diagram for of Cu-, Ag-, and Au-TPC complexes. Occupied and unoccupied MOs are indicated in black and blue, respectively. Note that the HOMO energy levels are similar. However, the LUMO energy levels vary across the three complexes as a result of varying levels of interaction between the corrole’s π-HOMO and the formally empty metal (d_x2-y2_) orbital. Reproduced from ref (13). Copyright 2017 American Chemical Society.

Herein, all samples were prepared under ultrahigh vacuum (UHV) conditions. The M[TPC] samples were sublimed from an alumina crucible heated by a tungsten filament. For Cu- and Au[TPC], a sublimation temperature of 250 °C was used, while a lower temperature of 200 °C was used for Ag[TPC]. UPS and IPES experiments were performed on two distinct instruments. For IPES, the substrate was cut from a Si wafer covered by an amorphous carbon thin film. For UPS, an Au(111) film grown on a mica substrate was used. The cleanliness of the substrate and the thickness of the sample film were both probed by X-ray photoelectron spectroscopy and Auger electron spectroscopy. The film thickness used was large enough (several nm) so to avert problems arising from interface interaction or band bending.

IPES experiments were carried out in UHV at ∼10^–10^ mbar base pressure. The measurements were performed in the isochromat mode, i.e., the incident electron kinetic energy was varied and emitted photons of fixed energy (9.7 eV) were collected by a band-pass photon analyzer consisting of a CaF_2_ entrance window and a Geiger-Müller detector.^61^ The incident electronic current was about 2 μA and the photon yield about 30–50 counts/s. The photon counts were normalized to the measured injected current. No significant sample degradation was observed when comparing the first and the last scan for each spectrum. The spectra were all referenced to the Fermi level measured on a clean Ta foil. The spectra were least-squares fitted with Gaussians with FWHM = 0.85 eV (in line with the energy resolution of the apparatus) along with an integral background.^72^ UPS measurements were performed with He I (hν = 21.22 eV) radiation from a HIS 13 discharge lamp from Scienta Omicron. The emitted photoelectrons were counted using an R3000 analyzer equipped with a microchannel plate detector. The resolution of the UPS measurements, determined from the width of the Fermi step on the metallic substrate, was 0.15 eV.

Figure 2 reports combined UPS-IPES-DFT spectra for the three M[TPC] thin films. On the filled-states side, essentially identical UPS spectra were measured, with very similar HOMO positions, consonant with similar electrochemical oxidation potentials for the three compounds.^35,62^ The overall line shape was well reproduced by DFT, as expected for weakly interacting units in a molecular film.^63^ On the other hand, IPES revealed major differences across the three molecules. From Cu through Ag to Au, a progressive shift to higher energies of the empty states was observed (Table 1), qualitatively mirroring the reduction potentials of the three complexes. Quantitatively, the solid-state band gap (*E*_g_) as measured by UPS-IPES can be related to the electrochemical HOMO–LUMO gap (*E*_ox-red_) with a normalization factor that accounts for different screening mechanisms (polarization) acting in the solid state vs in solution. As shown in Table 1, good agreement between the two energies was found with a normalization factor of 1.15.^58^ A least-squares fit procedure revealed the presence of three low-energy states (see Gaussian curves beneath the spectra in Figure 2) that could be rationalized with the help of DFT results.

**Figure 2:**
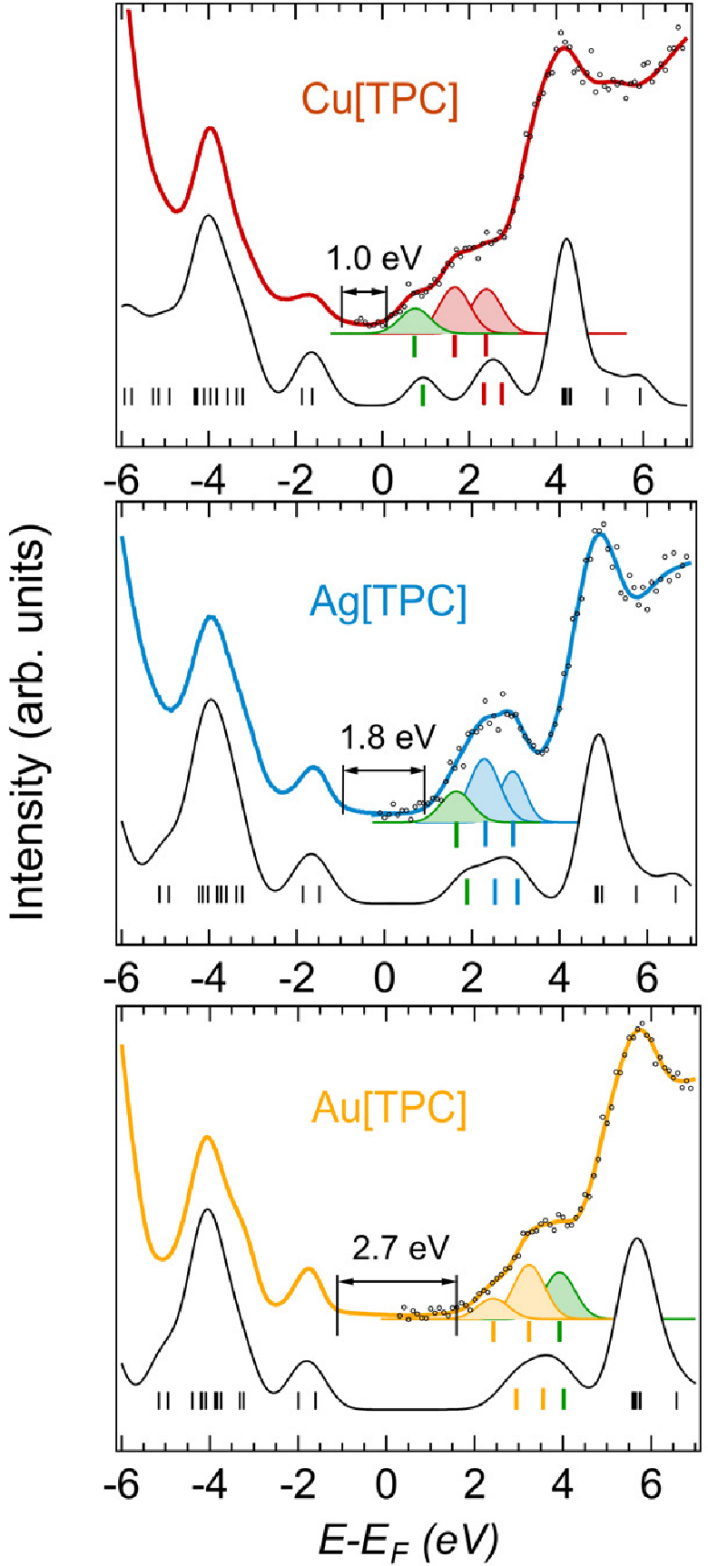
Experimental (upper curves) and calculated (lower curves) density of states (DOS) for the three M-TPC. Experimental curves are composed of UPS spectra (negative energies) measuring the occupied DOS up to the Fermi level (zero energy) and IPES spectra measuring the unoccupied DOS. For IPES, colored lines are the result of the fitting procedure to the experimental data (markers). All spectra are referenced to the Fermi level (*E*_F_). For each set of spectra, the three colored Gaussian curves correspond to the lowest unoccupied MO (LUMO), LUMO+1, and LUMO+2, resulting from the least-squares fitting of the IPES spectra. Their energy positions are highlighted by vertical bars. The experimental energy gap (*E*_g_) is indicated. Its value is determined as the energy difference between the HOMO and LUMO leading-edges’ intersections with the baseline (not shown).

**Table 1:** IPES Peak Positions Relative to the Fermi Level, UPS- and IPES-Derived Band Gaps (*E*_g_), and Electrochemical HOMO–LUMO Gaps (*E*_ox-red_)^a^

Compound	Peak a	Peak b	Peak c	*E*_g_	*E*_g_/1.15	*E*_ox-red_(35)
Cu[TPC]	0.74	1.66	2.39	1.0	0.85	0.96
Ag[TPC]	1.65	2.30	2.94	1.8	1.56	1.59
Au[TPC]	2.40	3.23	3.93	2.7	2.34	2.18

a All values are in eV. The peak labels a–c are ordered simply according to increasing energy relative to the Fermi level and do not have any connotations relative to the nature of the unoccupied state involved.

In the isolated molecules, the lowest-energy IPES feature can be assigned with a high degree of confidence from DFT calculations. Thus, the scalar-relativistic OLYP^64,65^-D3^66,67^/ZORA-STO-TZ2P method (which has been extensively tested by one of us^68−75^) yields gas-phase electron affinities^76−78^ that closely track the energies of the lowest-energy IPES feature (Table 2). For Cu[TPC], the LUMO corresponds to an antibonding combination of the corrole π-HOMO and the formally empty Cu 3d_x2-y2_ orbital, a consequence of the ligand noninnocence-driven saddled geometry of copper corroles (Figure 3). In the case of Ag[TPC], the saddling is much more muted so the corrole π-HOMO does not interact as much with the Ag 4d_x2-y2_ orbital and the LUMO corresponds to essentially the latter orbital (Figure 3; note the significantly smaller amplitudes at the corrole *meso* positions relative to Cu[TPC]). A very different scenario holds for Au[TPC]: relativistic effects^79−81^ raise the energy of the Au 5d_x2-y2_ to such a degree that it corresponds to the LUMO+2, while the LUMO corresponds to a pure corrole-based π-orbital (Figure 3).

**Table 2:** Selected All-Electron OLYP-D3/ZORA-STO-TZ2P Energetics (eV)^a^

	IP_1_	IP_2_	EA_1_		EA_2_		
Compound	vertical	vertical	vertical	adiabatic	vertical	adiabatic	Δ*E*_HOMO–LUMO_
Cu[TPC]	6.01 (^2^B)	6.18 (^2^A)	2.01 (^2^B)	2.18 (^2^B)	1.17 (^2^A)	1.24 (^2^A)	0.82
Ag[TPC]	5.96 (^2^B)	6.28 (^2^A)	1.38 (^2^B)	1.61 (^2^B)	1.28 (^2^A)	1.35 (^2^A)	1.19
Au[TPC]	6.01 (^2^B)	6.35 (^2^A)	1.14 (^2^A)	1.22 (^2^A)	0.77 (^2^B)	1.14 (^2^B)	1.75

a The calculations were carried out with a scalar-relativistic ZORA (Zeroth Order Regular Approximation to the Dirac equation)^59^ Hamiltonian, all-electron ZORA STO-TZ2P basis sets, fine integration grids and tight criteria for SCF and geometry optimization cycles, and *C*_2_ point group symmetry, all as implemented in the ADF program system.^60^ All IP and EA values were obtained via a ΔSCF procedure, i.e., as energy differences between initial and final states, with careful specification of electron occupancies in each irrep, where warranted. Note that a more positive electron affinity corresponds to a lower-energy LUMO. The HOMO-LUMO gaps (Δ*E*_HOMO–LUMO_) were obtained from Kohn-Sham orbital energies (see Figure 3).

**Figure 3:**
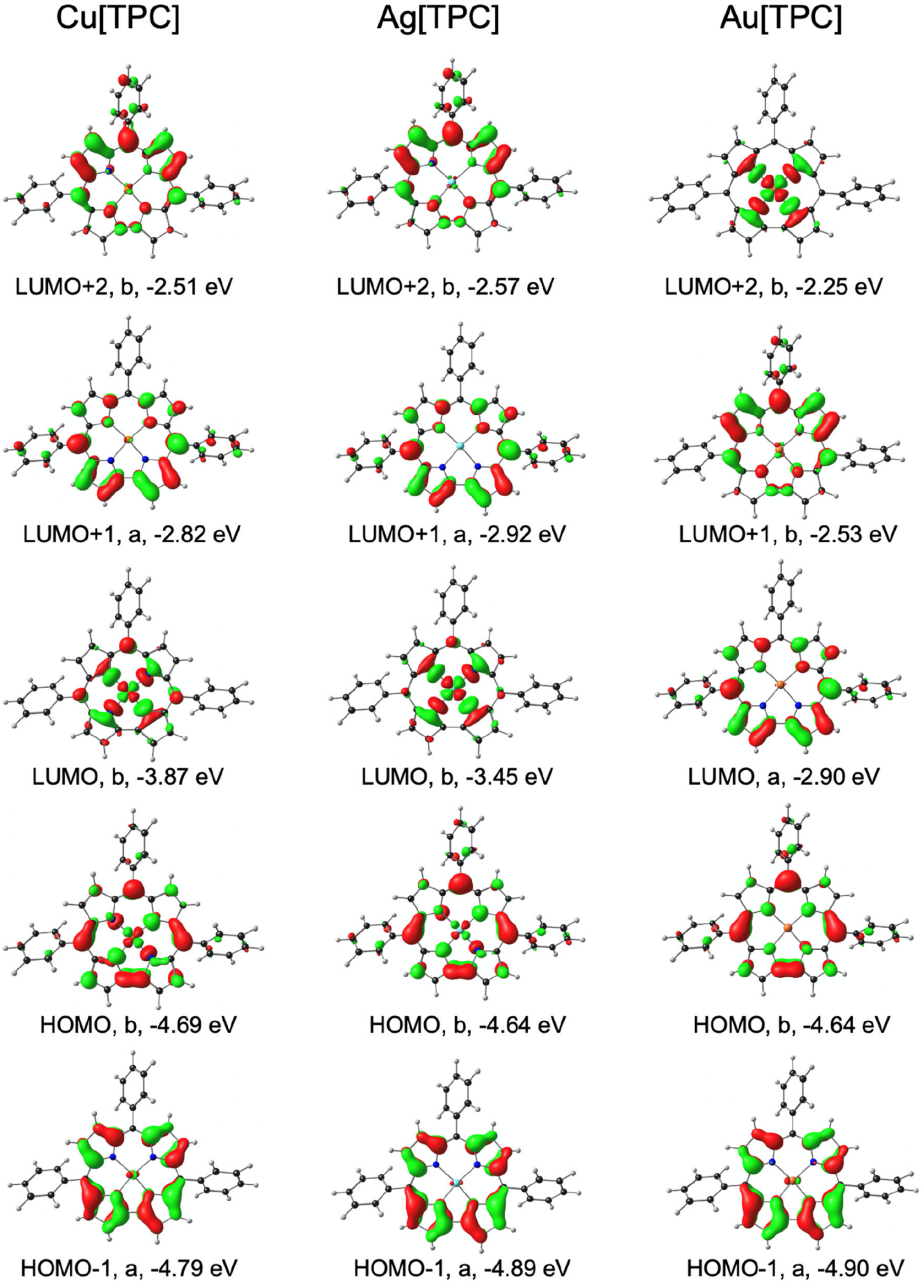
OLYP-D3/ZORA-STO-TZ2P frontier MOs of M[TPC], along with their *C*_2_ irreps and Kohn–Sham orbital energies.

Accordingly, in each panel of Figure 2, the three lowest unoccupied Kohn–Sham states are color-coded: the green bar, corresponding to the state carrying M(d_x2-y2_) character, moves to higher energy from Cu through Ag to Au. For Ag[TPC], the energy of the second IPES feature is very close to that of the first IPES feature of Au[TPC]. In light of the above discussion, it seems reasonable to assign this feature to a corrole-based LUMO. Indeed, the DFT-derived second electron affinities of both Cu[TPC] and Ag[TPC] are very close to the first electron affinity of Au[TPC] (Table 2). The assignment of the second IPES feature of Cu[TPC], however, remains somewhat uncertain. DFT calculations suggest that this feature should arise from an essentially corrole-based LUMO, but the energy (1.66 eV) seems unduly lower than that of an analogous feature for Ag- and Au[TPC].

In summary, an IPES study of coinage metal triphenylcorroles has uncovered major differences in the energetics of the unoccupied states for the three metals. While the results nicely mirror those obtained from electrochemistry and DFT calculations, they also afford additional insight. Thus, in the case of Ag[TPC], IPES appears to have yielded unique experimental data on the energetics of the LUMO and LUMO+1-derived anion states.^3−6^ Overall, the IPES results are consistent with the electroactive nature of copper corroles, such as in dioxygen reduction and evolution processes, relative to gold corroles. The latter are of great interest as triplet photosensitizers, especially in photomedicine, in applications such as oxygen sensing and photodynamic therapy.^82−85^ With continuing improvements in experimental methodology,^22^ the day may not be far when IPES enjoys a significantly wider range of applications to metalloporphyrinoids and other transition metal complexes, including catalysts and metallodrugs, and particularly inorganic polymers and other systems that are not readily studied with solution-phase techniques such as electrochemistry and optical spectroscopy.

## Data Availability

The data underlying this study are available in the published article and its Supporting Information.

## Supporting Information Available

Optimized DFT coordinates (PDF)
