# Supplementary material for: Inverse Photoemission Spectroscopy of Coinage Metal Corroles: Comparison with Solution-Phase Electrochemistry
Source: ACS Org Inorg Au. 2024 Jun 19;4(5):485–91. doi: 10.1021/acsorginorgau.4c00027 (PMC11450770; doi:10.1021/acsorginorgau.4c00027)
Supplement: Supplementary file 1 — gg4c00027_si_001.pdf [file gg4c00027_si_001.pdf]

Supporting information

Inverse Photoemission Spectroscopy of Coinage Metal Corroles: Comparison with Solution-Phase Electrochemistry

Luca Giovanelli,*[,c] Younal Ksari,[a] Hela Mrezguia,[a] Eric Salomon,[b]
Marco Minissale[b] Abraham B. Alemayehu[c] and Abhik Ghosh*[,c]

[a] Aix-Marseille Université, CNRS, IM2NP, Marseille, France.
[b] Aix-Marseille Université, CNRS, PIIM, Marseille, France.
[c] Department of Chemistry, UiT – The Arctic University of Norway, N-9037 Tromsø, Norway

Correspondence: luca.giovanelli@im2np.fr (LG), abhik.ghosh@uit.no (AG)

Optimized Cartesian coordinates (Å)

All compounds were optimized the ZORA Hamiltonian, the OLYP exchange-correlation functional augmented with D3 dispersion corrections, and all-electron ZORA/STO-TZ2P basis sets, all as implemented in the ADF program system. The structures were identified as minima via frequency analyses, which showed the absence of imaginary frequencies. ADF does not yield conventional total energies; interested readers are advised to generate them using the coordinates provided below and a quantum chemistry software of their choice.

Table of Contents

1. Cu[TPC], C_2 2
2. Ag[TPC], C_2 3
3. Au[TPC], C_2 4

1. Cu[TPC], C_2

Cu	0.000000000	0.000000000	0.078563000
N	1.401522000	-0.282811000	-1.174913000
N	-1.401522000	0.282811000	-1.174913000
N	-1.103908000	0.567456000	1.504273000
N	1.103908000	-0.567456000	1.504273000
C	2.629446000	-0.864198000	-0.899103000
C	3.205262000	-1.309198000	-2.134693000
C	2.318787000	-1.019912000	-3.138911000
C	1.179085000	-0.392902000	-2.537038000
C	0.000000000	0.000000000	-3.197767000
C	-1.179085000	0.392902000	-2.537038000
C	-2.318787000	1.019912000	-3.138911000
C	-3.205262000	1.309198000	-2.134693000
C	-2.629446000	0.864198000	-0.899103000
C	-3.199425000	1.044307000	0.386022000
C	-2.448677000	0.847143000	1.553296000
C	-2.848446000	0.783106000	2.930228000
C	-1.742160000	0.408034000	3.674544000
C	-0.667119000	0.239303000	2.758839000
C	0.667119000	-0.239303000	2.758839000
C	1.742160000	-0.408034000	3.674544000
C	2.848446000	-0.783106000	2.930228000
C	2.448677000	-0.847143000	1.553296000
C	3.199425000	-1.044307000	0.386022000
C	4.609689000	-1.471214000	0.496770000
C	4.972064000	-2.538704000	1.335710000
C	6.302512000	-2.938271000	1.445893000
C	7.297684000	-2.276129000	0.723460000
C	6.950535000	-1.213077000	-0.113500000
C	5.620133000	-0.816929000	-0.227687000
C	0.000000000	0.000000000	-4.679843000
C	0.986478000	0.695610000	-5.396117000
C	0.984122000	0.699579000	-6.789436000
C	0.000000000	0.000000000	-7.491618000
C	-0.984122000	-0.699579000	-6.789436000
C	-0.986478000	-0.695610000	-5.396117000
C	-4.609689000	1.471214000	0.496770000
C	-5.620133000	0.816929000	-0.227687000
C	-6.950535000	1.213077000	-0.113500000
C	-7.297684000	2.276129000	0.723460000
C	-6.302512000	2.938271000	1.445893000
C	-4.972064000	2.538704000	1.335710000
H	4.196127000	-3.059038000	1.888467000
H	6.562857000	-3.772418000	2.093665000
H	8.336390000	-2.585999000	0.811982000
H	7.719744000	-0.686107000	-0.673515000
H	5.350043000	0.015091000	-0.870420000
H	-1.748942000	-1.239211000	-4.846730000
H	1.748942000	1.239211000	-4.846730000

H	1.749809000	1.253044000	-7.328311000
H	0.000000000	0.000000000	-8.579092000
H	-1.749809000	-1.253044000	-7.328311000
H	-4.196127000	3.059038000	1.888467000
H	-6.562857000	3.772418000	2.093665000
H	-8.336390000	2.585999000	0.811982000
H	-7.719744000	0.686107000	-0.673515000
H	-5.350043000	-0.015091000	-0.870420000
H	-4.156298000	1.813654000	-2.217965000
H	-2.407725000	1.252945000	-4.189309000
H	2.407725000	-1.252945000	-4.189309000
H	4.156298000	-1.813654000	-2.217965000
H	3.856779000	-0.939230000	3.285591000
H	1.695513000	-0.246187000	4.743765000
H	-1.695513000	0.246187000	4.743765000
H	-3.856779000	0.939230000	3.285591000

2. Ag[TPC], C_2

Ag	0.000000000	0.000000000	0.102660000
N	1.441248000	-0.323389000	-1.218533000
N	-1.441248000	0.323389000	-1.218533000
N	-1.160021000	0.512119000	1.604850000
N	1.160021000	-0.512119000	1.604850000
C	2.656334000	-0.903334000	-0.897386000
C	3.215842000	-1.368736000	-2.139220000
C	2.328463000	-1.084777000	-3.149696000
C	1.185900000	-0.434355000	-2.567386000
C	0.000000000	0.000000000	-3.213750000
C	-1.185900000	0.434355000	-2.567386000
C	-2.328463000	1.084777000	-3.149696000
C	-3.215842000	1.368736000	-2.139220000
C	-2.656334000	0.903334000	-0.897386000
C	-3.213595000	1.034009000	0.405652000
C	-2.496975000	0.787033000	1.606315000
C	-2.895314000	0.723001000	2.985682000
C	-1.783797000	0.373601000	3.747757000
C	-0.684324000	0.211169000	2.857697000
C	0.684324000	-0.211169000	2.857697000
C	1.783797000	-0.373601000	3.747757000
C	2.895314000	-0.723001000	2.985682000
C	2.496975000	-0.787033000	1.606315000
C	3.213595000	-1.034009000	0.405652000
C	4.621254000	-1.478995000	0.515649000
C	4.978396000	-2.529483000	1.377255000
C	6.302741000	-2.949880000	1.478572000
C	7.298629000	-2.326981000	0.723669000
C	6.957746000	-1.280043000	-0.135457000
C	5.633413000	-0.861050000	-0.238007000
C	0.000000000	0.000000000	-4.697551000
C	1.017167000	0.648520000	-5.415662000

C	1.016799000	0.650135000	-6.808907000
C	0.000000000	0.000000000	-7.511397000
C	-1.016799000	-0.650135000	-6.808907000
C	-1.017167000	-0.648520000	-5.415662000
C	-4.621254000	1.478995000	0.515649000
C	-5.633413000	0.861050000	-0.238007000
C	-6.957746000	1.280043000	-0.135457000
C	-7.298629000	2.326981000	0.723669000
C	-6.302741000	2.949880000	1.478572000
C	-4.978396000	2.529483000	1.377255000
H	4.202442000	-3.022931000	1.954057000
H	6.557297000	-3.772258000	2.143452000
H	8.332507000	-2.654973000	0.804154000
H	7.727288000	-0.783205000	-0.721988000
H	5.369142000	-0.040198000	-0.897341000
H	-1.803980000	-1.157700000	-4.867453000
H	1.803980000	1.157700000	-4.867453000
H	1.808388000	1.165969000	-7.347904000
H	0.000000000	0.000000000	-8.598816000
H	-1.808388000	-1.165969000	-7.347904000
H	-4.202442000	3.022931000	1.954057000
H	-6.557297000	3.772258000	2.143452000
H	-8.332507000	2.654973000	0.804154000
H	-7.727288000	0.783205000	-0.721988000
H	-5.369142000	0.040198000	-0.897341000
H	-4.164505000	1.877322000	-2.227671000
H	-2.428579000	1.326293000	-4.197411000
H	2.428579000	-1.326293000	-4.197411000
H	4.164505000	-1.877322000	-2.227671000
H	3.900841000	-0.887593000	3.345523000
H	1.754877000	-0.237932000	4.821288000
H	-1.754877000	0.237932000	4.821288000
H	-3.900841000	0.887593000	3.345523000

3. Au[TPC], C_2

Au	0.000000000	0.000000000	0.101742000
N	1.394469000	-0.405628000	-1.224341000
N	-1.394469000	0.405628000	-1.224341000
N	-1.184984000	0.413338000	1.590090000
N	1.184984000	-0.413338000	1.590090000
C	2.662303000	-0.871010000	-0.906051000
C	3.286135000	-1.186634000	-2.162647000
C	2.402328000	-0.905568000	-3.175119000
C	1.192023000	-0.402676000	-2.585598000
C	0.000000000	0.000000000	-3.232658000
C	-1.192023000	0.402676000	-2.585598000
C	-2.402328000	0.905568000	-3.175119000
C	-3.286135000	1.186634000	-2.162647000
C	-2.662303000	0.871010000	-0.906051000
C	-3.207883000	1.032143000	0.392471000

C	-2.491789000	0.800314000	1.595332000
C	-2.858093000	0.858486000	2.982293000
C	-1.748605000	0.506902000	3.747249000
C	-0.680346000	0.216684000	2.855332000
C	0.680346000	-0.216684000	2.855332000
C	1.748605000	-0.506902000	3.747249000
C	2.858093000	-0.858486000	2.982293000
C	2.491789000	-0.800314000	1.595332000
C	3.207883000	-1.032143000	0.392471000
C	4.617851000	-1.477968000	0.504669000
C	4.952058000	-2.612265000	1.260711000
C	6.276271000	-3.033946000	1.365527000
C	7.292620000	-2.327855000	0.718865000
C	6.973314000	-1.194374000	-0.031288000
C	5.649188000	-0.773718000	-0.136311000
C	0.000000000	0.000000000	-4.718535000
C	0.945754000	0.747707000	-5.435722000
C	0.948322000	0.745964000	-6.829310000
C	0.000000000	0.000000000	-7.531952000
C	-0.948322000	-0.745964000	-6.829310000
C	-0.945754000	-0.747707000	-5.435722000
C	-4.617851000	1.477968000	0.504669000
C	-5.649188000	0.773718000	-0.136311000
C	-6.973314000	1.194374000	-0.031288000
C	-7.292620000	2.327855000	0.718865000
C	-6.276271000	3.033946000	1.365527000
C	-4.952058000	2.612265000	1.260711000
H	4.160110000	-3.166903000	1.754769000
H	6.514652000	-3.920698000	1.948583000
H	8.325877000	-2.656741000	0.801030000
H	7.758605000	-0.631680000	-0.531113000
H	5.400542000	0.112638000	-0.711933000
H	-1.677547000	-1.333591000	-4.887610000
H	1.677547000	1.333591000	-4.887610000
H	1.687030000	1.335222000	-7.368029000
H	0.000000000	0.000000000	-8.619396000
H	-1.687030000	-1.335222000	-7.368029000
H	-4.160110000	3.166903000	1.754769000
H	-6.514652000	3.920698000	1.948583000
H	-8.325877000	2.656741000	0.801030000
H	-7.758605000	0.631680000	-0.531113000
H	-5.400542000	-0.112638000	-0.711933000
H	-4.280384000	1.595206000	-2.260740000
H	-2.554173000	1.045043000	-4.234545000
H	2.554173000	-1.045043000	-4.234545000
H	4.280384000	-1.595206000	-2.260740000
H	3.838693000	-1.124239000	3.348410000
H	1.700835000	-0.454868000	4.826982000
H	-1.700835000	0.454868000	4.826982000
H	-3.838693000	1.124239000	3.348410000
